# ERα propelled aberrant global DNA hypermethylation by activating the DNMT1 gene to enhance anticancer drug resistance in human breast cancer cells

**DOI:** 10.18632/oncotarget.8038

**Published:** 2016-03-12

**Authors:** Xinxin Si, Yue Liu, Jinghuan Lv, Haijian Ding, Xin A. Zhang, Lipei Shao, Nan Yang, He Cheng, Luan Sun, Dongliang Zhu, Yin Yang, Andi Li, Xiao Han, Yujie Sun

**Affiliations:** ^1^ Key Laboratory of Human Functional Genomics of Jiangsu Province, Nanjing Medical University, Nanjing, Jiangsu, China; ^2^ Collaborative Innovation Center for Cancer Medicine, Jiangsu Key Lab of Cancer Biomarkers, Prevention and Treatment, Nanjing Medical University, Nanjing, Jiangsu, China; ^3^ State Key Laboratory of Reproductive Medicine, Nanjing Medical University, Nanjing, Jiangsu, China; ^4^ Department of Physiology and Stephenson Cancer Center, University of Oklahoma Health Sciences Center, Oklahoma City, Oklahoma, USA; ^5^ Department of Cell Biology, Nanjing Medical University, Nanjing, Jiangsu, China; ^6^ Department of Pathology, Municipal Hospital, Suzhou Hospital Affiliated to Nanjing Medical University, Suzhou, Jiangsu, China

**Keywords:** ERα, DNMT1, DNMT3b, global DNA hypermethylation, breast cancer chemoresistance

## Abstract

Drug-induced aberrant DNA methylation is the first identified epigenetic marker involved in chemotherapy resistance. Understanding how the aberrant DNA methylation is acquired would impact cancer treatment in theory and practice. In this study we systematically investigated whether and how ERα propelled aberrant global DNA hypermethylation in the context of breast cancer drug resistance. Our data demonstrated that anticancer drug paclitaxel (PTX) augmented ERα binding to the DNMT1 and DNMT3b promoters to activate DNMT1 and DNMT3b genes, enhancing the PTX resistance of breast cancer cells. In support of these observations, estrogen enhanced multi-drug resistance of breast cancer cells by up-regulation of DNMT1 and DNMT3b genes. Nevertheless, the aberrant global DNA hypermethylation was dominantly induced by ERα-activated-DNMT1, since DNMT1 over-expression significantly increased global DNA methylation and DNMT1 knockdown reversed the ERα-induced global DNA methylation. Altering DNMT3b expression had no detectable effect on global DNA methylation. Consistently, the expression level of DNMT1 was positively correlated with ERα in 78 breast cancer tissue samples shown by our immunohistochemistry (IHC) analysis and negatively correlated with relapse-free survival (RFS) and distance metastasis-free survival (DMFS) of ERα-positive breast cancer patients. This study provides a new perspective for understanding the mechanism underlying drug-resistance-facilitating aberrant DNA methylation in breast cancer and other estrogen dependent tumors.

## INTRODUCTION

Epigenetic instability plays an important role in cancer progression and metastasis [1-4]. Aberrant DNA methylation is the first identified epigenetic marker involved in chemotherapy resistance. Tumor cells exposed to toxic concentrations of commonly used cancer chemotherapy agents usually develop global DNA hypermethylation, both *in vitro* and *in vivo* [4-8]. This drug-induced DNA hypermethylation may create drug resistance by randomly inactivating genes whose products are required for chemotherapy agents to kill cancer cells [7, 9]. The DNA hypermethylation can result from aberrant expression of DNA methyltransferases (DNMTs) [10-13], primarily DNMT1, DNMT3a, and DNMT3b [14]. However, the mechanism that leads to the acquisition of aberrant DNMT expression in cancer drug resistance is poorly understood.

The functions of steroid hormones and their receptors in regulation of DNA methylation status have recently begun to draw attention [15-17]. Breast cancer is a highly hormone dependent cancer, with estrogen recognized as a classical etiological factor for breast carcinogenesis, development, and drug resistance. Estrogen mediates its biological effects in target tissues primarily by binding to specific intracellular receptors, the estrogen receptors ERα and ERβ [18]. Approximately 65% of human breast cancers express ERα [19] and around 40% of ERα-positive breast cancer patients inevitably relapse and have poor prognosis [20].

Chemotherapy is the usual treatment choice for early-stage invasive and advanced-stage breast cancer, before surgery or after surgery [21-22], as well as for recurrent and metastatic breast tumors [23-24]. However, chemoresistance is still a major obstacle limiting the success of breast cancer treatment. ERα has been confirmed to contribute to drug resistance of breast cancer, acting through mechanisms including inhibition of apoptosis and up-regulation of ABC transporters [25-26]. However, little is known about the functional relationship of ERα and drug-induced aberrant DNA methylation, although several reports have suggested ERα may be involved in regulation of DNMTs in lung cancer and endometrial adenocarcinoma [27-28]. Elucidation of a functional link between ERα and drug-induced hypermethylation will provide a special insight into mechanisms underlying drug-resistance-facilitating aberrant DNA methylation in breast cancer and other estrogen dependent tumors.

We have previously examined global DNA methylation alterations in ERα-positive and ERα-negative drug-resistant breast cancer cell lines based on analysis of the LINE-1 promoter methylation [29]. LINE-1, a type of repetitive element, comprises approximately 20% of human genome and has been usually used as a surrogate marker for estimating global DNA methylation [30-31]. We have found that paclitaxel-induced DNA hypermethylation is positively associated with the ERα expression status. ERα-positive drug-resistant MCF-7/PTX cells gain increased global DNA methylation (DNA hypermethylation), while ERα-negative drug-resistant MDA-MB-231/PTX cells lose global DNA methylation (DNA hypomethylation) compared with their parental cell lines cultured in parallel [29]. This finding suggests that ERα may be involved in drug-induced global DNA hypermethylation. Another indication of ERα involvement in epigenetic regulation from our previous work is that ERα significantly up-regulated DNMT1-luciferase reporter gene activity in breast cancer cells [29]. Genomatix software analysis (http://www.genomatix.de/index.html) showed that the promoter regions of DNMT1 and DNMT3b contained ERα binding sequences.

The aim of the present study is to determine whether and how ERα promotes aberrant global DNA hypermethylation in the context of breast cancer drug resistance. To this end we systematically investigated the role of ERα in regulation of DNMT gene activity and the resulting effect on global DNA methylation based on two PTX resistant breast cancer cell lines, MCF-7/PTX and ZR-75-1/PTX and their parental cell lines. The *in vitro* data were further evaluated in breast cancer tissue samples. Our data demonstrated that ERα propelled aberrant global DNA hypermethylation by activating the DNMT1 gene to enhance anticancer drug resistance in human breast cancer cells.

## RESULTS

### The expression level of ERα was positively correlated with DNMT1 and DNMT3b expression in breast cancer cells

**Figure 1:**
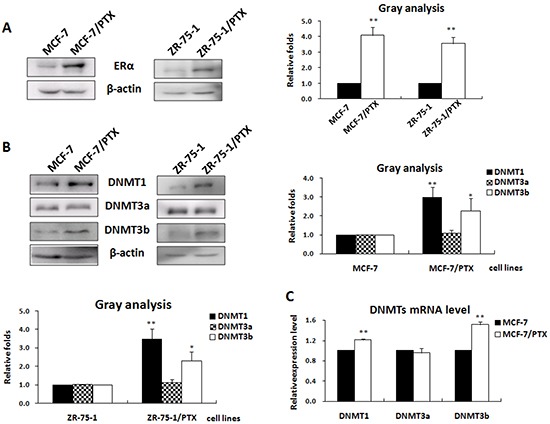
The expression of ERα was positively correlated with that of the DNMT1 and DNMT3b in breast cancer cell lines **A.** Western blot analysis of the ERα expression in PTX-resistant breast cancer cell lines and their paired parental cell lines (left); the histogram depicting the relative ERα protein levels (right). **B.** Western blot analysis of the DNMTs protein levels in the two pairs of PTX-resistant breast cancer cells (left); the histogram depicting the relative expression levels of DNMTs proteins (right). **C.** Real-time PCR was performed to check the DNMTs transcriptional products.

To determine the role of ERα in regulation of the DNMTs expression, we first examined the expression levels of ERα and the three DNMTs in the PTX-resistant MCF-7/PTX and ZR-75-1/PTX cell lines established in our laboratory. Western blot analysis showed that the expression of ERα, DNMT1, and DNMT3b was significantly increased in MCF-7/PTX and ZR-75-1/PTX cell lines, when compared with the paired parental MCF-7 and ZR-75-1 cell lines (Figure 1A & 1B). By contrast, the expression level of DNMT3a was the same in the drug-resistant breast cancer cell lines and the parental controls. The increased expression of DNMT1 and DNMT3b was, at least in part, a result of transcription up-regulation of these two genes, as the mRNA levels were correspondingly increased in these two drug resistant breast cancer cell lines (Figure 1C). The positive correlation between ERα and DNMT1 and DNMT3b expression suggested that ERα might be involved in up-regulation of the DNMTs in breast cancer drug response.

### ERα up regulated the expression of DNMT1 and DNMT3b in ERα-positive breast cancer cells

**Figure 2:**
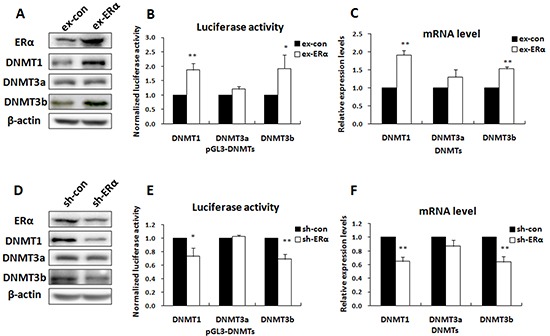
ERα activated DNMT1 and DNMT3b genes in ERα-positive breast cancer cells **A.** Western blot was performed to check the expression levels of ERα and DNMTs in MCF-7 cells transfected with ERα expression vectors. **B.** Luciferase reporter assay showed that over-expression of ERα enhanced the promoter activities of DNMT1 and DNMT3b, but not DNMT3a, in MCF-7 cells. **C.** Real-time PCR showed that over-expression of ERα up regulated the intracellular mRNA levels of DNMT1 and DNMT3b, but not DNMT3a, in MCF-7 cells. **D.** Western blot was performed to check the expression levels of ERα and DNMTs in MCF-7/PTX cells transfected with ERα-shRNA plasmids. **E.** Luciferase reporter assays showed that knockdown of ERα reduced DNMT1 and DNMT3b promoter activities in MCF-7/PTX cells. **F.** Real-time PCR showed that knockdown of ERα reduced the DNMT1 and DNMT3b intracellular mRNA levels in MCF-7/PTX cells.

To determine the functional role of ERα in up-regulation of DNMT1 and DNMT3b expression, we tested whether change in ERα expression altered the promoter activity of the DNMT genes by reporter gene analysis and real time PCR. Luciferase reporter vectors containing the DNMT1, DNMT3b, or DNMT3a promoters were prepared and transfected into MCF-7 cells where ERα was over-expressed. The transfection efficiency was confirmed by Western blot analysis (Figure 2A). The results showed that introduction of ERα into MCF-7 cells significantly increased the DNMT1 and DNMT3b reporter gene activities (Figure 2B), while only slightly affecting the DNMT3a reporter gene activity. Consistently, the cellular mRNA and protein levels of DNMT1 and DNMT3b, but not DNMT3a, were elevated by ERα over-expression (Figure 2C & 2A).

The promoting effect of ERα on DNMT1 and DNMT3b expression was further confirmed by RNA interference experiments. ERα expression was knocked down in MCF-7/PTX cells with plasmids expressing short hairpin RNAs (shRNA) and the targeting efficiency was confirmed by Western blot analysis (Figure 2D). As expected, ERα knockdown attenuated the DNMT1 and DNMT3b reporter gene activities (Figure 2E) and reduced the cellular mRNA and protein levels (Figure 2F & 2D). These results verified that ERα was able to promote DNMT1 and DNMT3b expression in breast cancer cells.

### ERα binding to the DNMT1 and DNMT3b promoters was significantly increased in PTX-resistant breast cancer cells

**Figure 3:**
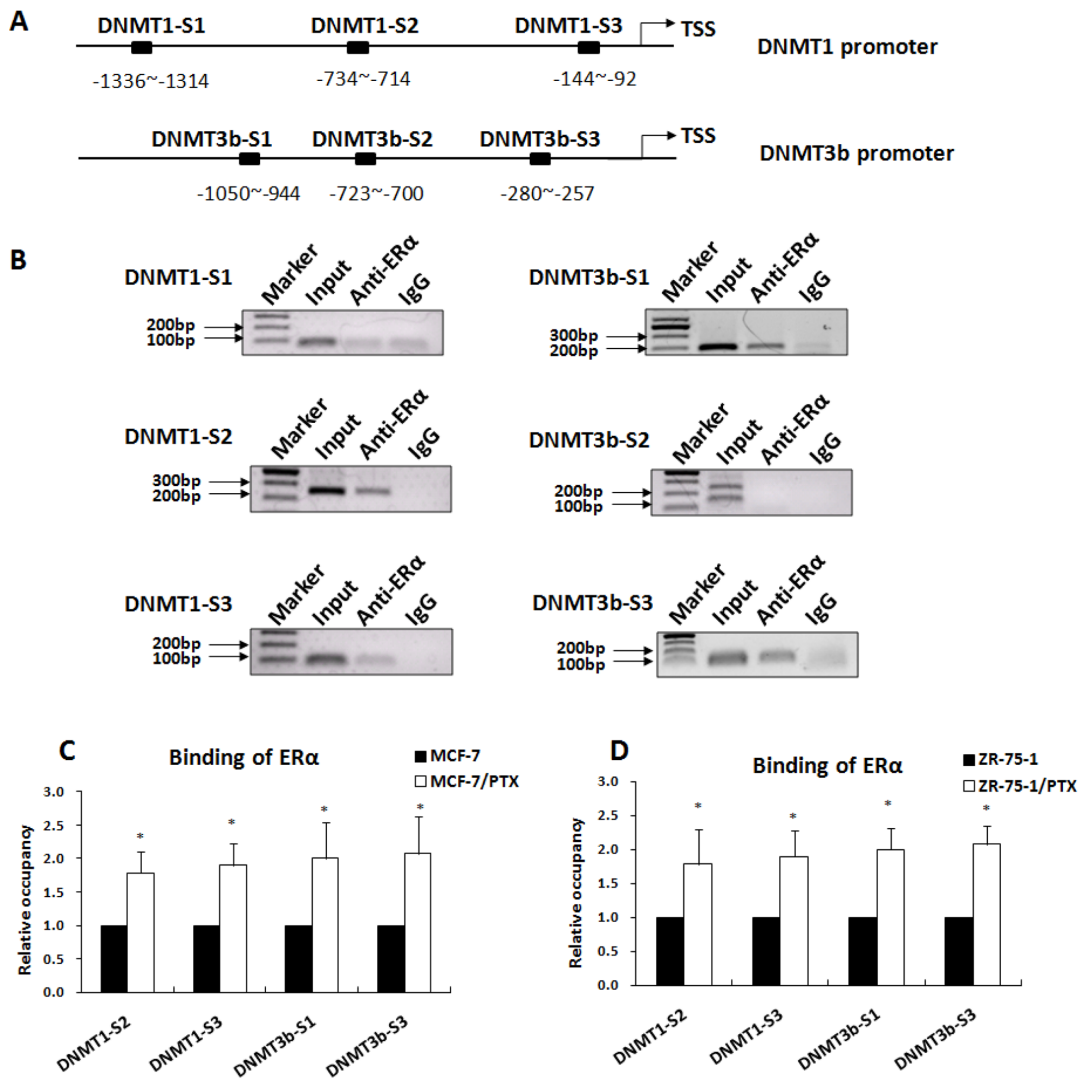
ERα occupancy on the DNMT1 and DNMT3b promoters was significantly increased in PTX-resistant breast cancer cells **A.** Diagram of the ERα binding sites in the human DNMT1 and DNMT3b gene promoters indicated by bioinformatics analysis. **B.** ChIP assay revealed that the DNMT1-S2 and DNMT1-S3 and the DNMT3b-S1 and DNMT3b-S3 were immunoprecipitated with ERα antibody, confirming ERα binds to theses sequences in breast cancer cells. **C.** qChIP assay indicated that the bindings of ERα to the DNMT1 and DNMT3b promoters were significantly increased in MCF-7/PTX cells when compared with the parental MCF-7 cells. **D.** The qChIP assay was repeated in ZR-75-1/PTX cells and similar results were obtained, indicating that anticancer drug exposure enhanced binding of ERα to the DNMT1 and DNMT3b promoters.

ERα is known to function as transcription factor by directly binding to a specific estrogen response element (ERE) within the promoter or by interacting with other transcription factors that bind to the promoter [32-33]. Bioinformatics analysis revealed that the DNMT1 and DNMT3b promoters contained several potential ERα binding sequences (Figure 3A). We tested whether DNMT1 and DNMT3b were the direct target genes of ERα by performing ChIP assays using an anti-ERα antibody to examine ERα binding to the gene promoters. As indicated in Figure 3B, the DNMT1-S2, DNMT1-S3, DNMT3b-S1, and DNMT3b-S3 sequences were specifically immunoprecipitated with anti-ERα antibody, indicating that ERα bound to these sequences *in vivo*. No specific precipitates were detected for DNMT1-S1 and DNMT3b-S2.

The functional relationship between the ERα binding and breast cancer drug resistance was further evaluated by qChIP assay. Our data showed that ERα binding to the DNMT1 and DNMT3b promoter regions was significantly increased in MCF-7/PTX drug-resistant cells when compared to the parental MCF-7 cells (Figure 3C). These results were further confirmed in ZR-75-1/PTX and ZR-75-1 breast cancer cells (Figure 3D). These findings suggested that ERα activated DNMT1 and DNMT3b expression by direct binding to the gene promoters in the response of breast cancer cells to anticancer drugs.

### DNMT1 or DNMT3b expression enhanced drug resistance of breast cancer cells and was negatively correlated with the prognosis of breast cancer patients

**Figure 4:**
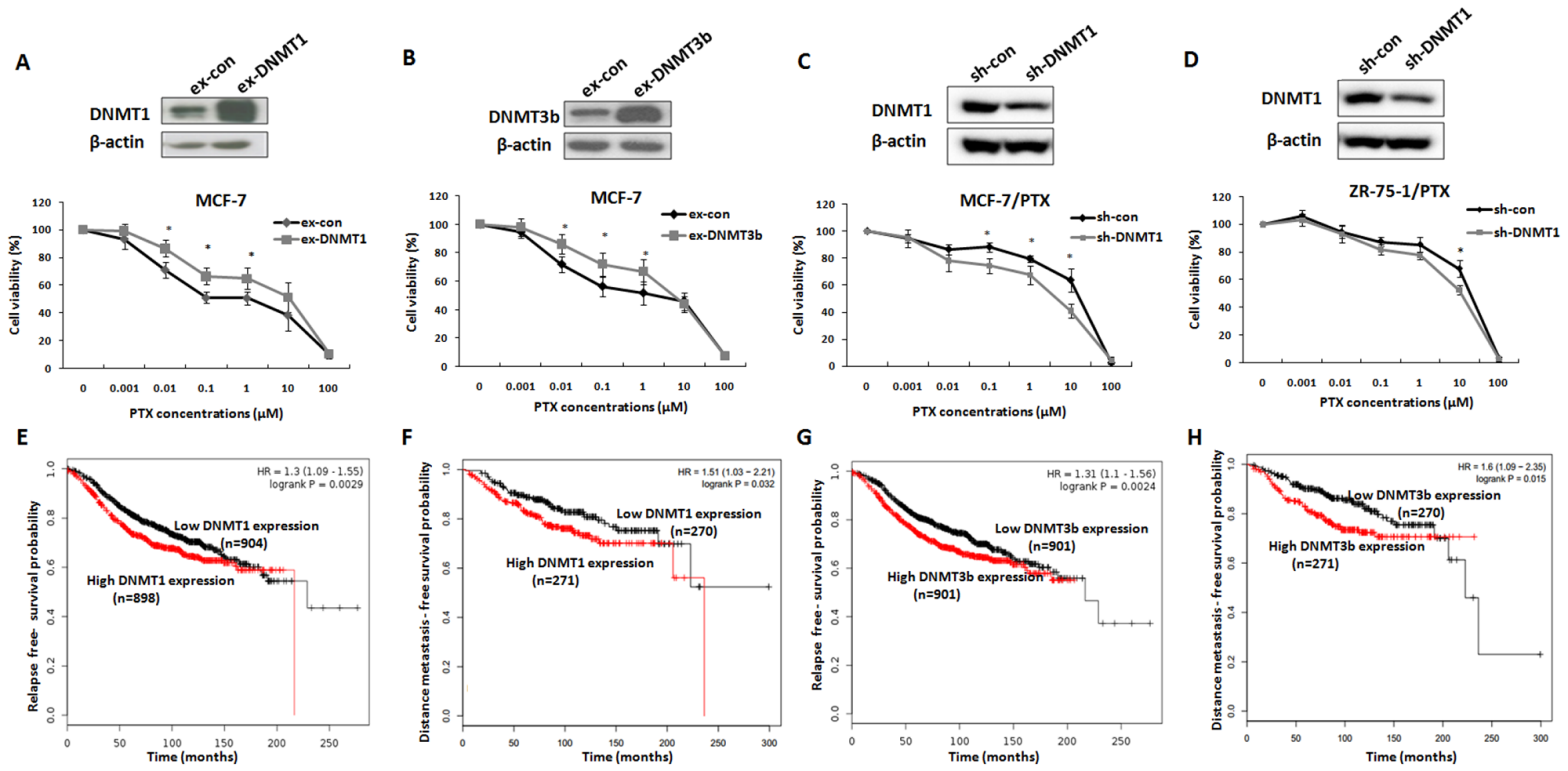
DNMT1 or DNMT3b expression enhanced drug resistance of breast cancer cells and was negatively correlated with the prognosis of breast cancer patients **A.** Western blot analysis of DNMT1 expression in MCF-7 cells transiently transfected with DNMT1 expression plasmids (upper panel). MTT assay indicated that over-expression of DNMT1 increased viability of breast cancer cells under the stress of PTX treatment (lower panel). **B.** Similar experiments were performed to test the effect of DNMT3b on the response of breast cancer cells to PTX. DNMT3b over-expression increased the cell survival in the presence of PTX. **C, D.** Western blot analysis of DNMT1 expression in MCF-7/PTX (upper left) or ZR-75-1/PTX (upper right) cells transiently transfected with DNMT1-shRNA plasmids (upper). MTT assay was performed to determine cell viabilities of MCF-7/PTX (lower left) or ZR-75-1/PTX cells (lower right) treated with PTX at different concentrations. **E, F.** Kaplan-Meier analysis revealed negative correlation between DNMT1 and RFS and DMFS of ERα-positive breast cancer patients. **G, H.** Kaplan-Meier analysis displayed the similar results regarding the correlation between DNMT3b and the RFS and DMFS.

Subsequent to determination of the ERα activating role in DNMTs genes, we evaluated the role of ERα-induced DNMTs up-regulation in acquired drug resistance of breast cancer cells by testing whether alteration of DNMT1 and DNMT3b expression change drug sensitivity of breast cancer cells. MCF-7 cells were transfected with DNMT1 or DNMT3b expression plasmids and the transfection efficiencies were confirmed by Western blot analysis (Figure 4A & 4B). At 24 h after transfection, the cells were treated with PTX at different concentrations for 48 h and then harvested for viability tests using MTT assays. The over-expression of either DNMT1 or DNMT3b increased cell viability when compared with the control (Figure 4A & 4B), indicating that increased DNMT1 or DNMT3b expression promoted cell survival in the presence of PTX. These results were further confirmed by knockdown of DNMT1 in MCF-7/PTX and ZR-75-1/PTX drug resistant breast cancer cell lines. As expected, reduction of DNMT1 expression by RNAi could partly reversed drug resistance phenotype of these two PTX-resistant breast cancer cell lines (Figure 4C & 4D).

The clinical significance of the DNMT1 and DNMT3b high expression was evaluated in ERα-positive breast cancer patients by Kaplan-Meier Plotter analysis (http://kmplot.com/breast/). As shown in Figure 4E & 4F, patients with high DNMT1 expression in their breast cancer samples had lower relapse-free survival (RFS) and distance metastasis-free survival (DMFS) than those with low DNMT1 expression in the samples. Similar results were obtained for the DNMT3b expression (Figure 4G & 4H). The negative correlation between the DNMT1 and DNMT3b expression levels and the prognosis of ERα-positive breast cancer patients was consistent with the observations in breast cancer cell lines, suggesting that high expression of DNMT1 and DNMT3b has a detrimental effect on breast cancer drug response.

### Both DNMT1 and DNMT3b were downstream target genes of ERα and involved in ERα-induced drug resistance

**Figure 5:**
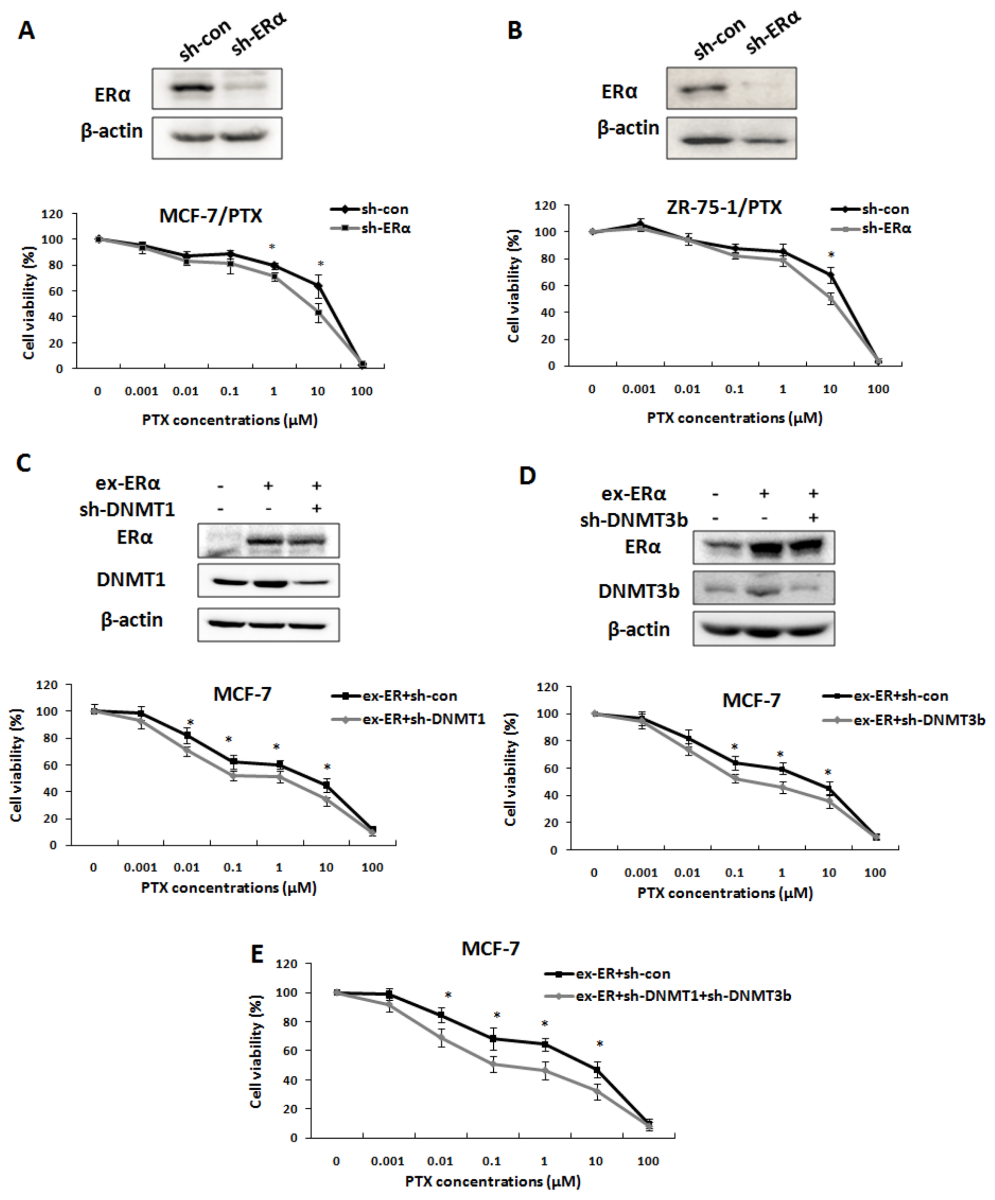
DNMT1 and DNMT3b were downstream target genes of ERα in ERα-mediated chemoresistance **A, B.** Western blot was performed to determine the ERα knockdown efficiency in MCF-7/PTX (upper left) or ZR-75-1/PTX cells (upper right) transfected with ERα-shRNA. MTT assay showed that knockdown of ERα partly restored the sensitivity of PTX drug resistant breast cancer cells (lower panel), indicating ERα contributed to breast cancer drug resistance. **C, D.** Western blot was performed to check the transfection efficiencies in MCF-7 cells transfected with ERα expression plasmids together with either DNMT1-shRNA plasmids (C, upper panel) or DNMT3b-shRNA plasmids (D, upper panel). MTT assays were performed to examine the cell viability. Knockdown of DNMT1 (C, lower panel) or DNMT3b (D, lower panel) reduced the effect of ERα over-expression on the drug resistance of the MCF-7 cells. **E.** MTT assays showed that double knockdown of DNMT1 and DNMT3b restricted the effect of ERα over-expression on drug resistance more than the single DNMT knockdown.

ERα is known to be an important contributor to breast cancer chemoresistance [25-26]. To determine the functional link of ERα, DNMTs and breast cancer drug resistance, we first confirmed the effect of ERα on the drug resistance phenotype with our PTX-resistant breast cancer cell lines by RNAi experiments. As expected, knockdown of ERα in MCF-7/PTX and ZR-75-1/PTX cell cells partly reversed the drug resistance phenotype. The IC50 values decreased from 16.61 ± 2.78 μM to 7.42 ± 0.57 μM and 17.23 ± 2.09 μM to 6.16 ± 2.34 μM, respectively (Figure 5A & 5B). Then we addressed whether DNMT1 and DNMT3b were the downstream target genes of ERα in breast cancer drug resistance. MCF-7 cells were co-transfected with ERα expression plasmid together with either DNMT1-shRNA or DNMT3b-shRNA. The targeting efficiencies were confirmed by Western blot (Figure 5C & 5D). At 24 h after transfection, the cells were treated with PTX at different concentrations for 48 h and then harvested for viability tests. The MTT assays showed that DNMT1 or DNMT3b knockdown partly restrained the effect of ERα over-expression and sensitized the MCF-7 cells to PTX (Figure 5C & 5D). The IC50 values significantly decreased from 1.57 ± 0.41 μM to 0.52 ± 0.09 μM and from 1.52 ± 0.1 μM to 0.46 ± 0.07 μM for the DNMT1 and DNMT3b knockdown, respectively. Furthermore, double knockdown of DNMT1 and DNMT3b restrained the effect of ERα over-expression more efficiently than the DNMT1 or DNMT3b single knockdown. The IC50 value decreased from 1.6 ± 0.08 μM to 0.35 ± 0.05 μM (Figure 5E). These results strongly indicated that DNMT1 and DNMT3b were downstream target genes of ERα and involved in ERα-induced drug resistance.

### Estrogen increased DNMT1 and DNMT3b expression and enhanced the multi-drug resistance of ERα-positive breast cancer cells

**Figure 6:**
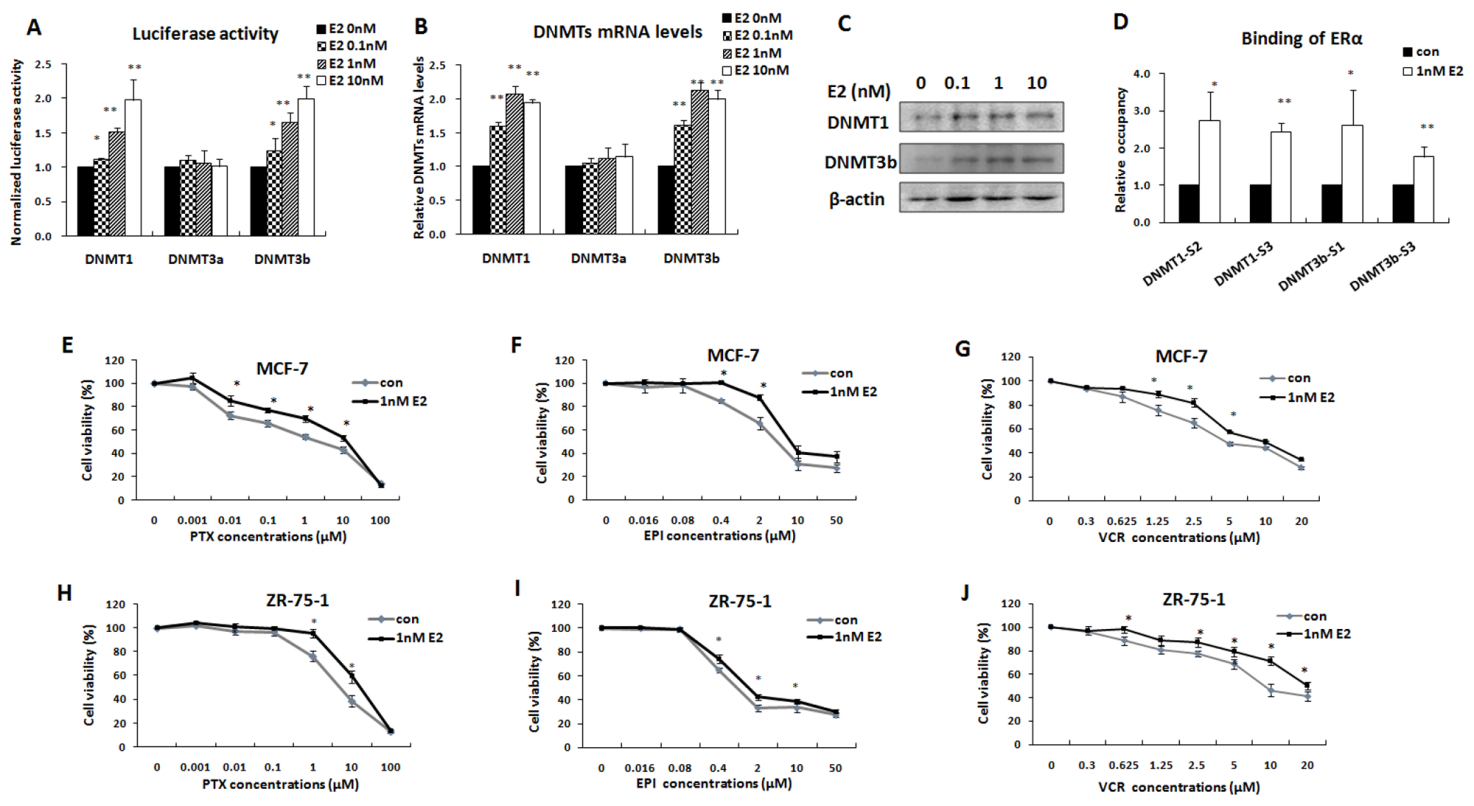
Estrogen increased DNMT1 and DNMT3b expression and enhanced the multi-drug resistance of ERα-positive breast cancer cells **A.** Luciferase reporter assay was performed to detect DNMTs promoter activities following treatment with graded concentrations of estrogen. **B.** Real-time PCR was performed to detect transcriptional levels of DNMTs in MCF-7 cells treated with estrogen. **C.** Western blot was performed to detect the expression levels of DNMT1 and DNMT3b in MCF-7 cells treated with estrogen. **D.** qChIP assay confirmed that estrogen treatment increased the bindings of ERα to DNMT1 and DNMT3b promoters in MCF-7 cells. **E–J.** MTT assay showed that estrogen increased the resistance of MCF-7 and ZR-75-1 cells to multi anticancer drugs, including PTX (E, H), EPI (F, I) and VCR (G, J).

The functional relationship between ERα, DNMT1, and DNMT3b was further validated by treating MCF-7 cells with estrogen (E2), a ligand of ERα. As indicated in Figure 6A, E2 dose-dependently increased the DNMT1 and DNMT3b reporter gene activities and up regulated both mRNA and protein levels of DNMT1 and DNMT3b, as confirmed by real time PCR and Western blot analysis (Figure 6B & 6C). This stimulatory effect was induced by activation of ERα, as qChIP assay demonstrated that E2 treatment significantly increased ERα binding on the DNMT1 and DNMT3b promoters (Figure 6D).

Subsequently, we tested whether E2 enhanced the multi-drug resistance of ERα-positive breast cancer cells. MCF-7 cells were pretreated with 1 nM E2 for 24 h and then treated with different chemotherapeutic agents, PTX, EPI, or VCR. 48 h after drug treatment, cells were harvested for testing viability with MTT assays. The results showed that the cells pretreated with E2 were more resistant to these anticancer drugs than the control (Figure 6E–6G). These results were further confirmed in ZR-75-1 cells (Figure 6H–6J). These data, together with those already described, strongly indicated that ERα activated-DNMTs promoted multi-drug resistance of ERα-positive breast cancer cells.

### ERα-activated DNMT1 induced the global DNA methylation level dominantly

**Figure 7:**
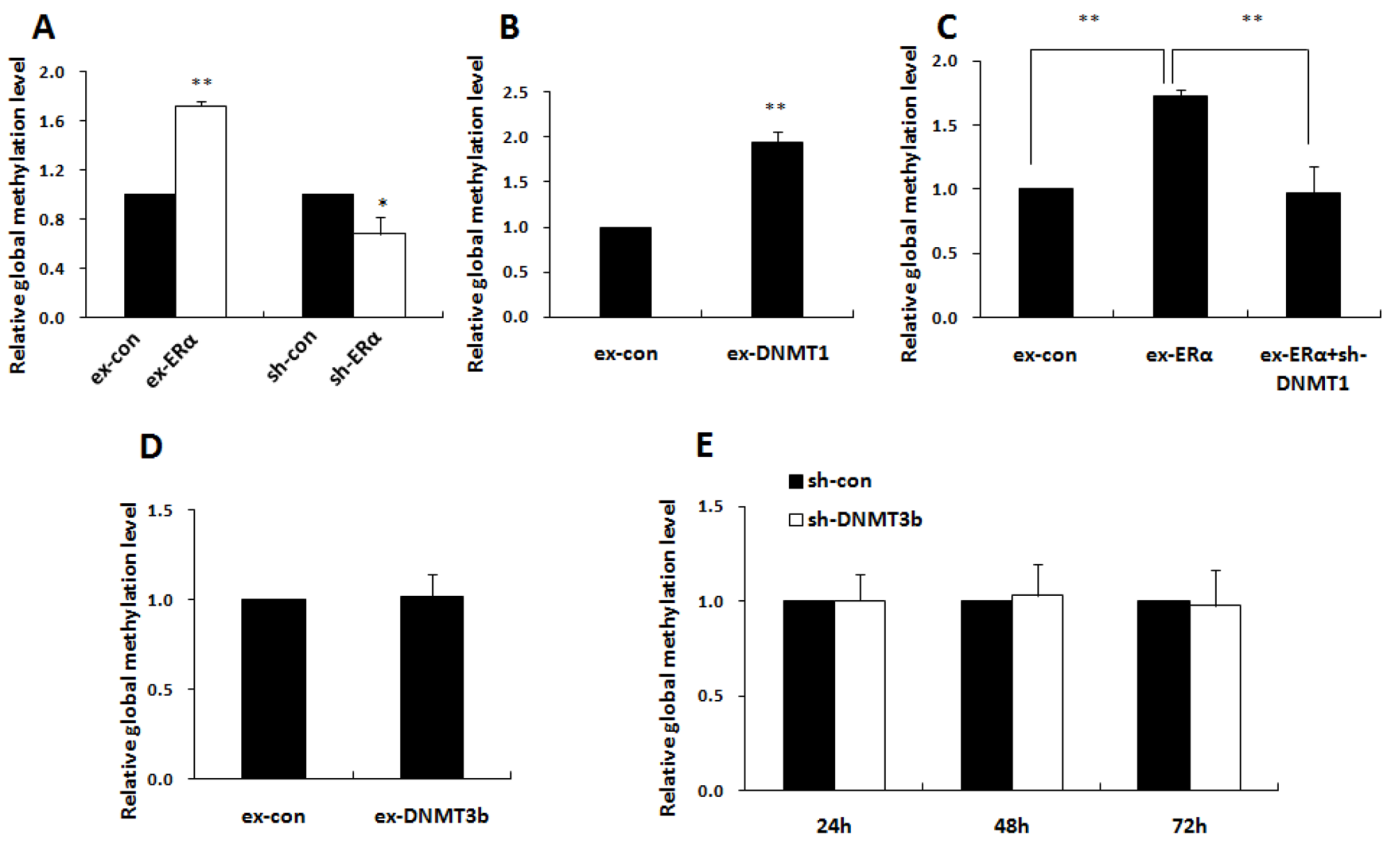
ERα elevated the global DNA methylation level through DNMT1 Quantitative methylation-sensitive PCR (qMSP) was performed to detect the genomic DNA methylation levels. **A.** qMSP showed that global methylation level was increased in MCF-7 cells transfected with ERα expression plasmids and was decreased in MCF-7/PTX cells transfected with ERα-shRNA plasmids when compared with their controls. **B.** qMSP showed that global methylation level was increased in MCF-7 cells transfected with DNMT1 expression plasmids compared with the control. **C.** qMSP showed that knockdown of DNMT1 in MCF-7 cells significantly restrained the ERα-induced global hypermethylation. **D, E.** qMSP showed that over-expression or knockdown of DNMT3b had no detectable effect on global DNA methylation level.

LINE-1 is a type of repetitive element that comprises approximately 20% of the human genome. Its methylation status closely parallels the overall global methylation level, so it is considered as a valid surrogate marker for estimating global DNA methylation [30-31]. Subsequent to identification of ERα function in activating DNMT1 and DNMT3b expression, we tested the effects of ERα on genome-wide methylation level and its relation to the specific DNMT in MCF-7 cells by determination of LINE-1 methylation levels with methylation-sensitive PCR (MSP). As expected, introduction of ERα into MCF-7 cells significantly increased the global DNA methylation level, while ERα knockdown attenuated the global methylation level (Figure 7A).

**Figure 8:**
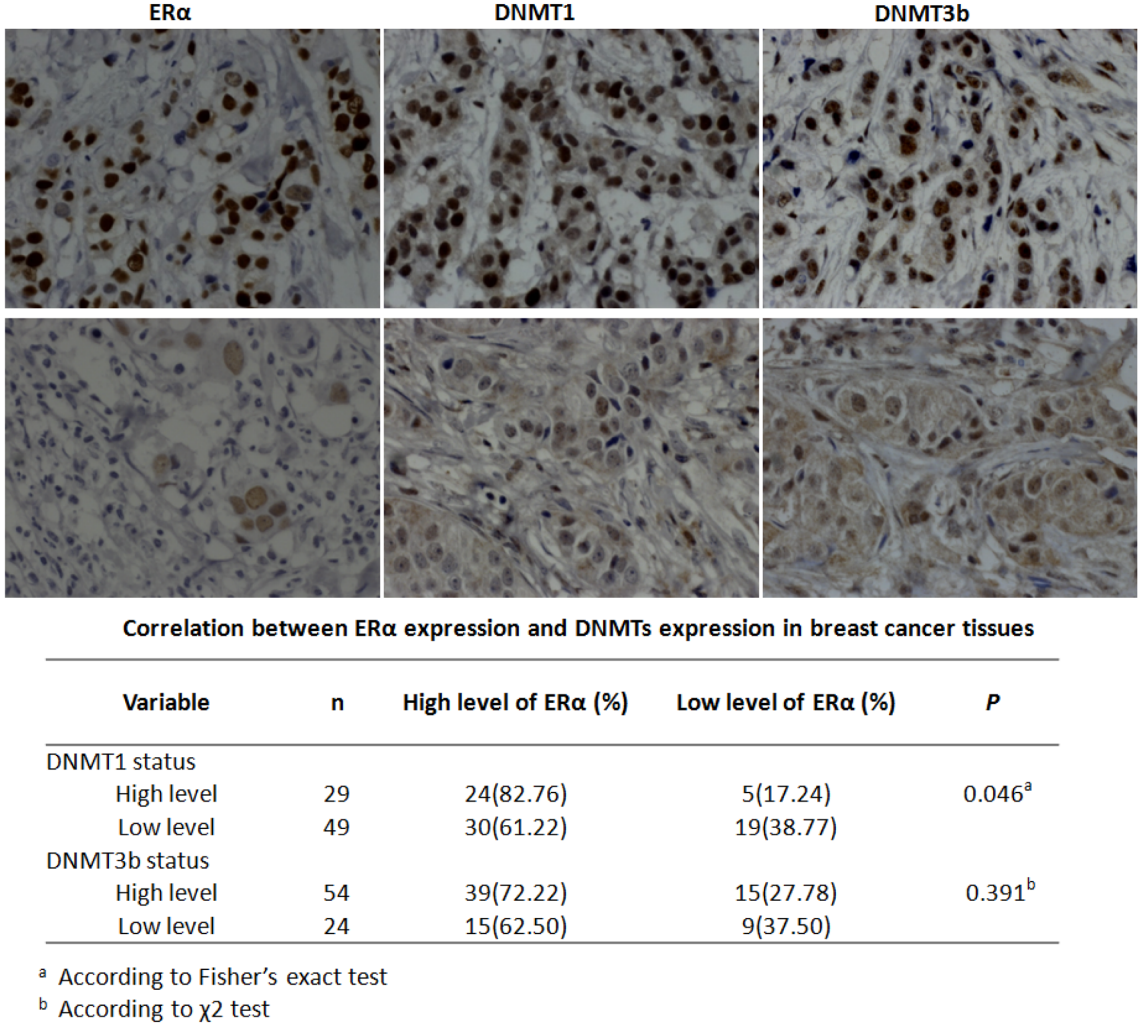
ERα expression was positively correlated with DNMT1 expression in breast cancer patients Representative immunohistochemical staining pictures of ERα, DNMT1 and DNMT3b in breast cancer tissues. The upper panel represented the strong positive staining and the lower panel represented the weak positive staining. The level of ERα in breast cancer tissues showed a statistically positive correlation with DNMT1, while no significant correlation with DNMT3b was observed.

Notably, DNMT1 over-expression doubled the global DNA methylation level compared with the control and DNMT1 knockdown reversed ERα-induced global hypermethylation (Figure 7B & 7C). By contrast, alteration of the DNMT3b expression had no detectable effect on the global DNA methylation (Figure 7D & 7E). These findings suggested that ERα induced global DNA methylation dominantly by activation of DNMT1 in breast cancer cells. The notion was supported by the positive correlation between DNMT1 and ERα expression (P=0.046) detected by our immunohistochemical analysis in 78 breast cancer tissues samples (Figure 8). No significant correlation between ERα and DNMT3b was observed (Figure 8).

## DISCUSSION

Aberrant DNA methylation is the known epigenetic marker involved in chemotherapy resistance. It can be resulted from abnormal expression of DNMTs [10-13]. In this study we systematically investigated whether and how ERα regulated DNMTs to facilitate drug resistance of breast cancer cells. Our data demonstrated that ERα increased the drug-induced global DNA hypermethylation through activation of the DNMT1 gene to enhance the anticancer drug resistance of breast cancer cells. Consistently, the DNMT1 expression was positively correlated with ERα expression in breast cancer tissues and negatively correlated with RFS and DMFS of ERα-positive breast cancer patients.

### ERα propelled drug-resistance-facilitating global DNA hypermethylation by activation of the DNMT1 gene in breast cancer cells

Several studies have suggested a role of estrogen in regulation of DNMTs; however, the results were discrepant. For example, estrogen was reported to increase DNMT3b expression in endometrial adenocarcinoma cells [28], but decrease DNMT3b transcription in an endometrial explant culture [34]. Estrogen treatment down regulated DNMT1 expression in lung cancer [27], but had no effect on DNMT1 in endometrial adenocarcinoma [28, 34]. Furthermore, little is known whether and how estrogen/ERα is involved in drug-induced aberrant DNA methylation. Our results strongly confirm that ERα can activate DNMT1 and DNMT3b genes by direct binding to the gene promoters in breast cancer cells. Estrogen enhanced multi-drug resistance of breast cancer cells by up-regulating DNMT1 and DNMT3b expression. These results are in contrast with those observed in lung cancer and endometrial adenocarcinoma [27-28, 34] and indicate that ERα is an activator for DNMT1 and DNMT3b genes in breast cancer cells. The discrepancy between the previously reported work and our results may mainly reflect the tissue specific function of ERα in regulating DNMTs expression.

It is notable that over-expression of DNMT1 alone doubles the global DNA methylation level in breast cancer cell lines examined and knockdown of DNMT1 significantly blocks the ERα-induced global DNA hypermethylation. Nevertheless, altering DNMT3b expression had no detectable effect on the global DNA methylation in the breast cancer cells. We speculate that ERα-activated-DNMT1 pathway dominantly propels the drug-induced global DNA hypermethylation in breast cancer, although the effects of DNMT3a/3b cannot be fully excluded in this experimental system and remains to be tested further. This notion is also supported by our previous observations that only DNMT1 expression was positively correlated with global DNA methylation level in two PTX-resistant breast cancer cell lines, MCF-7/PTX and MDA-MB-231/PTX. No significant correlation was detected in the case of DNMT3a and DNMT3b [29]. Further support is from our IHC analysis of 78 breast cancer tissue samples. DNMT1 expression is confirmed to be positively correlated with ERα expression in the breast cancer tissues. The Kaplan-Meier Plotter analysis indicates that DNMT1 expression was negatively correlated with RFS and DMFS of ERα-positive breast cancer patients. The data *in vitro* and *in vivo* together support the propelling role of ERα-activated-DNMT1 pathway in drug-resistance-facilitating aberrant global DNA hypermethylation.

Unexpectedly, we do not observe the significant correlation between DNMT3b and ERα expression in the 78 breast tissue samples, which is inconsistent with the observation *in vitro*. This discrepancy might be resulted from high heterogeneity of breast cancer. It deserves further investigating with more breast cancer cell lines and tissue samples, since DNMT3b expression is shown to be negatively correlated with RFS and DMFS of ERα-positive breast cancer patients by the Kaplan-Meier Plotter analysis.

The identification of ERα-DNMT1-global DNA hypermethylation is also informative regarding the distinct function of individual DNMT in DNA methylation. It implies that DNMT1 plays a significant role in de novo DNA methylation in breast cancer cells in addition to its function in maintaining DNA methylation. This is in line with the recent notion that DNMT1 has a considerable de novo methylation activity [35]. Thus, investigating the distinct role of DNMT1 in drug-induced global DNA hypermethylation and its relation to the other DNMTs will provide new clues for understand complex mechanisms of DNA methylation.

### ERα could epigenetically regulate multi-drug resistance of breast cancer cells through inducing aberrant DNA methylation

Aberrant DNA methylation has an important impact on gene expression. Global DNA hypermethylation may randomly inactivate genes whose products are required for chemotherapy agents to kill cancer cells [7]. In addition to global DNA hypermethylation, some genes may specifically undergo de novo methylation, leading to lack of specific gene products required for killing cancer cells [36-37]. We speculate that ERα facilitates drug resistance mainly through randomly inactivating genes required for killing breast cancer cells in the case of its epigenetic regulation, since ERα-activated-DNMT1 was dominantly involved in drug-induced global DNA hypermethylation. Extensive study to find out the genes that can be inactivated by ERα-DNMT1-propelled global DNA hypermethylation and to elucidate their functions in drug response will be very significant.

ERα-DNMT3b may catalyze specific gene de novo methylation, as it seems not to be involved in global DNA hypermethylation. Many genes, including MTSS1, RASSF1a, APC, TBX18, p16, and HOXB13, have been confirmed as targets of DNMT3b [38-40]. However, the functional relationship between ERα and DNMT3b needs to be further determined in more breast cancer tissue samples as described above.

The epigenetic function of ERα in breast cancer drug resistance implies that selective estrogen receptor down-regulators (SERDs) could have a potential role in inhibiting anticancer drug-induced aberrant DNA methylation. Since DNMTs inhibitors have the potential risk in inducing carcinogenesis, our results are illuminating regarding epigenetic correction of cancer drug resistant phenotype. It deserves testing whether combination of anticancer drugs with SERDs could inhibit anticancer drug-induced aberrant DNMT expression and DNA hypermethylation. This will provide insight into development of new chemotherapy strategies for breast cancer and other estrogen dependent cancers.

### Maintaining the balance between ERα and DNMTs expression might be a promising strategy for treatment of ERα-positive breast cancer

ERα is encoded by the ESR1 gene, and most studies have focused on regulation of ESR1 expression by DNMTs [41-43]. DNMT1, DNMT3a, and DNMT3b all function as suppressor of ERα expression by increasing methylation level of the ESR1 promoter. Specific difference in ESR1 gene methylation has been found between normal and breast tumor-adjacent tissues, and between ERα-positive and ERα-negative breast cancer cells [41]. In contrast, the knowledge of ERα function in regulating DNMTs expression is very sparse and discrepant. Our present study demonstrates that ERα is an activator of DNMT1 and DNMT3b in breast cancer cells. We suppose there may be a feedback loop to maintain a balance between ERα and DNMTs in normal breast cells. Disruption of this balance might result in abnormal ERα expression and aberrant DNA methylation in estrogen-dependent cancer cells. From this point of view, restoring the ERα-DNMTs balance might be a promising strategy for breast cancer treatment.

Taken together, the present study investigates the acquisition of aberrant DNA methylation from a new perspective and reveals an intrinsic link between ERα and drug-induced aberrant DNA methylation in the context of anticancer drug resistance. This study will provide valuable clues for understanding the mechanism underlying drug-resistance-facilitating aberrant DNA methylation in breast cancer and other estrogen dependent tumors.

## MATERIALS AND METHODS

### Cell culture, reagents, and plasmids

Human breast cancer cell lines MCF-7 and ZR-75-1 were obtained from ATCC. The PTX-resistant cell lines MCF-7/PTX and ZR-75-1/PTX were established by pulse selection with PTX. MCF-7 and MCF-7/PTX cells were cultured in MEM supplemented with 10% FBS, insulin (0.2 U/ml), 100 U/ml penicillin and 100 U/ml streptomycin, whereas ZR-75-1 and ZR-75-1/PTX cells were cultured in DMEM containing 10% FBS, 100 U/ml penicillin and 100 U/ml streptomycin. To determine the effect of estrogen (E2) on DNMTs expression, the cells were cultured in phenol red-free medium for 24 h before the application of E2 treatment. Thereafter, the cells were cultured in the absence or in the presence of E2 at various concentrations, and then were used for subsequent real-time PCR, Western blot, or MTT analysis. The vehicle control for E2 was an equal volume of ethanol. Estrogen was purchased from Sigma (St Louis, MO).

The ERα expression vector, DNMT1 expression vector, ERα-shRNA vector, DNMT1-shRNA vector, and DNMT1 promoter luciferase reporter vector were described in our previously work [29]. DNMT3a and DNMT3b promoter luciferase reporter vectors were cloned into pGL3-basic vector. DNMT3a and DNMT3b promoter sequences were amplified by PCR. The primers for DNMT3a were 5′-KpnI-GCCGGTACCATGCGCCATGACACCCAGC-3′ (forward), 5′-XhoI-CCGCTCGAGCTACCTGGCGCTGCT TC-3′ (reverse). The primers for DNMT3b were 5′-KpnI-CGGGGTACCTCCAACAACAATATGCCCC-3′ (forward), 5′-HindIII-CCCAAGCTTCGATCGCCGAGCTAGGTTT-3′ (reverse). DNMT3b expression vector containing DNMT3b full length coding sequence was constructed based on pcDNA3.0. DNMT3b-specific shRNA sequences were synthesized and inserted into the pRNAT-H1.1/neo vector. The DNMT3b-shRNA targeting sequence was: 5′- AGGTAGGAAAGTACGTCGC -3′.

### Clinical samples and IHC staining

78 paraffin-embedded ERα-positive breast cancer specimens were obtained from Suzhou Hospital Affiliated to Nanjing Medical University. The clinical data of the patients were collected including their gender, age, pathological subtype, lymph node metastasis, etc. This study was approved by the ethics committees of Nanjing Medical University. The tumor samples were immunostained with ERα (abcam), DNMT1 (abcam) and DNMT3b (abcam) antibodies. The IHC procedure and scoring of protein expression were performed as previously described [44]. Immunohistochemical signals were scored by three independent investigators in a double-blind way.

### ChIP assay

Chromatin immunoprecipitation (ChIP) assays were performed using the ChIP assay kit as described in the manufacturer (Millipore). Briefly, 1×10^7^ cells were fixed in 1% formaldehyde at 37°C for 10 min. The cross-linking was stopped by 1/20V of 2.5 M glycine. Then cells were lysed and sonicated into 200-1000 bp fragments and incubated with ERα antibody (Millipore) and IgG (Millipore) overnight at 4°C. Reversal of cross-linking was carried out at 65°C for 5 h, followed by DNA isolation. The input genomic DNA and the immunoprecipitated DNA was then amplified by PCR. The PCR products were subjected to gel electrophoresis, stained with ethidium bromide, and analyzed on a Molecular Imager Gel Doc XR System (Bio-Rad).

For quantitative analysis of ChIP products, real-time PCR was carried out to determine fold enrichment relative to input DNA. Primers for detection of the estrogen responsive element (ERE) region in the DNMT1 and DNMT3b promoters were listed in Supplementary Table S1. Ct values were calculated using the formula: ΔCt=Ct_sample_−Ct_input_, and ΔΔCt=ΔCt_experiment sample_ - Ct_negative control_. The fold increase of ERα binding was then calculated using the 2^−ΔΔCt^ method.

### DNA extraction and quantitative methylation-sensitive PCR (qMSP)

Total DNA was extracted using a Multisource Genomic DNA Miniprep Kit (Axygen) according to the manufacturer's protocol. A 1 μg amount of genomic DNA from each sample was modified with sodium bisulfite using the CpGenomeTM DNA Modification Kit (Chemicon). β-actin was used to normalize DNA inputs; a region of β-actin devoid of any CpG dinucleotide was amplified. The primer sequences were listed in Supplementary Table S1.

### Luciferase reporter assay

Cells were seeded in 12-well plates and co-transfected with a series of plasmids on the following day, including firefly reporter constructs containing target gene promoters, Renilla expressing plasmid, and ERα expression plasmid or control plasmid. Firefly luciferase activity, normalized to Renilla luciferase activity, was measured 48 h after the initiation of transfection by the Dual Luciferase Assay System (Promega).

### Survival curves

Cells were seeded at a density of 8000 cells per well in 96-well plates. On the following day, cells were treated with graded concentrations of paclitaxel (PTX), epirubicin (EPI), or vincristine (VCR). At the end of the culture, cell viability was measured using the MTT assay as previously described [29]. All measurements were done in triplicate.

### RNA extraction and quantitative real-time PCR assay

Total RNA was extracted using Trizol reagent (Invitrogen) according to the manufacturer's protocol. To prepare cDNA, 1 μg of total RNA was reverse-transcribed according to Roche manufacturer's instructions. Quantitative real-time PCR was carried out on the Light Cycler System using the double-strand DNA binding dye SYBR Green for the detection of PCR products. The following thermal cycling conditions were used: denaturation, 95°C for 10 min, followed by 40 cycles of denaturation at 95°C for 15 s, annealing at 60°C for 15 s, and extension at 72°C for 15 s. The primer sequences were listed in Supplementary Table S1.

### Western blot assay

Total cellular protein extracts were obtained and were separated on 10% SDS-polyacrylamide gel and transferred to PVDF membranes (Bio-Rad). After blocking in 5% skimmed milk for 1 h, membranes were incubated with a primary antibody overnight at 4°C. Membranes were washed with 3 times for 10 min in Tris-Buffered Saline with Tween-20 (TBST) and incubated with a HRP-conjugated secondary antibody (R&D) for 1 h at room temperature. After washing 3 times for 10 min in TBST, the membranes were developed with an ECL detection system. Quantification was performed using Quantity One (Bio-Rad). Antibodies against DNMT1 were purchased from Cell Signaling Technology, anti-DNMT3a was purchased from Santa Cruz, anti-DNMT3b was obtained from Abcam, and anti-ERα was from Santa Cruz; anti-β-actin was obtained from Sigma-Aldrich.

### Statistical analysis

All experiments were repeated three times. The results are presented as the mean ± SD. Data were analyzed using Student's t test to determine the level of significance between control and treatment groups. The χ^2^ test was used to determine the correlation between ERα and DNMTs in the breast cancer tissues. *P* < 0.05 was considered to be statistically significant.

## SUPPLEMENTARY TABLE


