# Supplementary material for: ERα propelled aberrant global DNA hypermethylation by activating the DNMT1 gene to enhance anticancer drug resistance in human breast cancer cells
Source: Oncotarget. 2016 Mar 12;7(15):20966–80. doi: 10.18632/oncotarget.8038 (PMC4991505; doi:10.18632/oncotarget.8038)
Supplement: Supplementary file 1 [file oncotarget-07-20966-s001.pdf]

SUPPLEMENTARY TABLE

Supplementary Table S1: Primers used in the present study

	Gene	Primer Sequence
RT-PCR	Actin-F	TCATGAAGTGTGACGTGGACAT
	Actin-R	CTCAGGAGGAGCAATGATCTTG
	DNMT1-F	CCTGAGGCCTTCACGTTCAA
	DNMT1-R	ACTTGTGGGTGTTCTCAGGC
	DNMT3a-F	CAGCGTCACACAGAAGCATATC
	DNMT3a-R	ACCACATTCTCAAAGA GCCAGA
	DNMT3b-F	GGATGAAGATCAGAGCCGAGAA
	DNMT3b-R	CAGGCACTCCACACAGAAACAC
qMSP	Actin-F	TGGTGATGGAGGAGGTTTAGTAAG
	Actin-R	AACCAATAAAACCTACTCCTCCCTTAA
	LINE1-F	GTCGAATAGGAAT AGTTTCGG
	LINE1R	ACTCCCTAACCCCTTACGCT
ChIP	DNMT1-1F	CTGGTTACGGTGGCTTAG
	DNMT1-1R	CTCCTAGCCTCAAGCAGT
	DNMT1-2F	TAACCTCAAGCGATCCTC
	DNMT1-2R	TGTGCCCATAGTCCAAAC
	DNMT1-3F	GCTTCTCGCTGCTTTATCC
	DNMT1-3R	CACGGTTTGCTGGGAGGT
	DNMT3b-1F	TGGAGTCACGGGAAAAGCC
	DNMT3b-1R	ACCTTAACCCCTCTCTGCCT
	DNMT3b-2F	GGAGACAGACCACAAGCAG
	DNMT3b-2R	ACTGACTGAGGGAGAAGC
	DNMT3b-3F	TGGGGGATCAGAAGCCCTAA
	DNMT3b-3R	TCTCCGTTCGGGTTGAAAGG
